# The Association of Diabetes Mellitus with Clinical Outcomes after Coronary Stenting: A Meta-Analysis

**DOI:** 10.1371/journal.pone.0072710

**Published:** 2013-09-16

**Authors:** Shan-Yu Qin, You Zhou, Hai-Xing Jiang, Bang-Li Hu, Lin Tao, Min-zhi Xie

**Affiliations:** 1 The First Affiliated Hospital of Guangxi Medical University, Nanning, Guangxi, PR China; 2 Minerva Foundation Institute for Medical Research, Helsinki, Finland; University of Padova, Medical School, Italy

## Abstract

**Background:**

Previous studies have shown inconsistent results on the association between diabetes mellitus (DM) and some clinical outcomes. We conducted a meta-analysis of observational studies to assess effect of DM on clinical outcomes after coronary stenting.

**Methods:**

We searched for studies without language restriction in PubMed, Embase and Cochrane library prior to 2012. The clinical outcomes including in-stent restenosis (ISR), major adverse cardiac events (MACE), stent thrombosis (ST), target lesion revascularization (TLR) and target vessel revascularization (TVR). Adjusted odds ratio (OR), and the corresponding 95% confidence interval (95% CI) was summarized.

**Results:**

55 studies involving 128,084 total patients (38,416 DM patients and 89,668 controls) were eligible for our analysis. Overall, there were significant associations between DM and ISR (OR = 1.70, 95% CI: 1.53–1.89, I^2^ = 0.0%), MACE (OR = 1.54, 95% CI: 1.36–1.73, I^2^ = 29.0%), ST (OR = 2.01, 95% CI: 1.36–2.97, I^2^ = 47.7%), TLR (OR = 1.46, 95% CI: 1.26–1.68, I^2^ = 43.3%) as well as TVR (OR = 1.33, 95% CI: 1.17–1.51, I^2^ = 48.3). Subgroup analysis showed that the associations were similar between BMS and DES implantation. Moreover, there was no significant association in the ST subgroup after 1–3 years follow-up.

**Conclusions:**

Our meta-analysis suggests that after coronary stent implantation, DM is associated with ISR, MACE, ST, TLR and TVR. DM appears to be a vital risk factor of these clinical outcomes.

## Introduction

As one of the most common non-communicable diseases in the world, diabetes mellitus (DM) has a profound impact on the development and progression of coronary artery disease (CAD) [1]–[3]. At present, percutaneous coronary intervention (PCI), including bare metal stent (BMS) and drug-eluting stent (DES) implantation, is a major and powerful revascularization strategy to treat the stenotic coronary arteries in CAD patients. Despite rapid development of revascularization techniques and adjunct antithrombotic therapies in the modern society, recent findings show that patients have increased rates of negative clinical outcomes compared with those without DM [4]–[6].

Although several studies reported that DM was an independent risk factor of clinical outcomes after coronary stenting [7]–[10], some other studies failed to identify the association in patients after DES implantation [11], [12]. Additionally, several meta-analyses [13], [14] and clinical trials [15], [16] showed inconsistent results which might be caused by 1) uncontrolled or incomplete control for confounding 2) relatively small sample size. Hence, the current findings on associations between DM and a number of clinical outcomes after stenting remain obscure. By using meta-analysis, we sought to accurately evaluate the relationships between DM and clinical outcomes.

## Materials and Methods

### Search strategy

In order to identify all the studies which examined the association between DM and the clinical outcomes after coronary stenting, we conducted a meta-analysis according to the guidelines of Preferred Reporting Items for Systematic Reviews and Meta-Analyses (PRISMA) [17]. We systematically searched Cochrane clinical trials database, Medline (PubMed) and EMBASE prior to June, 2012 following search terms: “diabetes mellitus” or “DM”, “stent”, “restenosis”, “major adverse cardiac events” or “MACE”, “thrombosis”, “target lesion revascularization” or “TLR”, “target vessel revascularization” or “TVR”. The search was not restricted by language or publication status. And the references of all retrieved publications were searched again to trace additional relevant studies. Moreover, the relevant review articles and their references were checked. In cases of multiple publications of the same or overlapping cohorts, the most recent ones with largest sample size were selected. At least two independent reviewers screened potentially relevant articles. Disagreements were resolved by discussion or upon consensus from the third reviewer.

### Inclusion and exclusion criteria

Studies that we identified should meet the following criteria: (1) the study design must be an observational study in human beings; (2) the study must have investigated the association between DM and the clinical outcomes after coronary stenting; (3) the study must have provided data of the associations between DM and clinical outcomes [the adjusted odds ratio (OR) and 95% confidence interval (95% CI)] after multivariate analysis. (4) ISR was defined as ≥50% diameter stenosis of the culprit lesion by quantitative coronary analysis. MACE varied slightly among a few studies, but generally it should consist of cardiac death, myocardial infarction and repeat revascularization. ST was defined as angiographic proven thrombus or total occlusion within the stent vessel at the time of clinically driven angiography for ischemia. TLR was defined as repeat PCI performed to revascularize the index lesion. TVR was defined as any PCI to revascularize the target vessel. (5) The follow-up duration must be at least 6 months. Excluded criteria were: laboratory studies, review articles, animal studies and the follow-up period shorter than 6 months.

### Data Extraction and quality assessment

Two blinded reviewers independently performed data extraction. Disagreements between the reviewers were resolved through discussion or by the third reviewer. The extracted data included: (1) first author's last name, the publication year, origin of the studied population; (2) characteristics of the study population, (3) stent types, duration of follow-up; (4) study design; (5) adjustment of confounding factors.

Two blinded reviewers independently performed the quality assessment. The quality of non-randomized observational studies was assessed by reporting the key components of study designs in accordance with guidelines of the Meta-analysis of Observational Studies in Epidemiology (MOOSE) group [18]. The quality of randomized controlled trials was evaluated according to the Cochrane Collaboration [19] by estimating the risk of selection, performance, detection and attrition bias (expressed as low risk of bias [A], moderate risk of bias [B], high risk of bias [C], or incomplete reporting leading to inability to ensure the underlying risk of bias [D]).

### Statistical analysis

Software STATA version 11.2 (Stata Corporation, College Station, TX, USA) was used for all analyses. Data were expressed as OR and 95% CI. The individual estimates of the log OR with its standard error from each study were synthesized to obtain the summary estimate of the OR by using inverse variance weighted method.

We assessed the heterogeneity between eligible studies by the Cochran Q test. We considered *P* values less than 0.10 as an indicator of significant heterogeneity because of the low statistical power. We also used the inconsistency index I^2^ to quantify heterogeneity [20]. The difference between subgroups was further measured by interaction test [21].

Funnel plot were constructed to assess publication bias by using Egger's linear regression test [22]. *P* values less than 0.05 indicated significant publication bias.

## Results

### Literature search

The primary literature search retrieved 1,018 records. After screening according to title, abstract and full text, 55 studies were finally selected including 128,084 total patients (38,416 DM patients and 89,668 controls. 29 studies (See supplement references in File S1) were prospective design, 18 were retrospective design, 8 were RCT. 21 studies were registered in clinical data base. The flow summary of selection process is presented in Figure S1.

### Studies characteristics and quality assessment

All the association data was adjusted according to confounding factors. All the studies clearly stated that the included and excluded criteria of subjects for the coronary stent implantation. The procedure of PCI with stent implantation and drug treatment of pre- and post procedure was also clearly stated. The diagnosis of DM was based on patients' history, or the intensively treated with insulin, or an oral antidiabetic agent, or patients had an abnormal blood glycemic level by overnight fasting and glycemic tolerance test according to the World Health Organization criteria. Patient demographics of each study are listed in Table 1,2,3,4,5, S1–S5 in File S1. The quality assessments of the studies are presented in Table S6–S7 in File S1.

**Table 1:** Characteristics of 20 studies investigating ISR in the meta-analysis.

Study	Year	Design	Stent	Age	Male	Total Patients	DM patients	FU	OR(95%CI)
Amano T(S1)	2006	Prospective	BMS	65	124(79.5)	156	59	6 m	2.93(1.11–7.73)
Ari H(S2)	2010	Prospective	B/D	60.3	80(76.2)	105	12	6 m	1.84(0.64–5.27)
Chen YL(S3)	2009	Retrospective	BMS	61	499(81.9)	609	197	6 m	1.831(1.274–2.633)
Ferrero V(S4)	2003	Retrospective	BMS	66	710(82.7)	858	162	6 m	1.5(1.2–1.8)
									1.6(1.2–2)
Hong SN(S5)	2006	Retrospective	BMS	56	87(72.5)	120	28	6 m	1.771(0.939–9.34)
Hong YJ(S6)	2007	Prospective	B/D	61	181(72.7)	249	70	6 m	2.494(0.714–7.342)
Ijsselmuiden AJJ(S7)	2003	RCT	BMS	61	324(81.0)	400	32	6 m	2.35(1.2–4.6)
Ino Y(S8)	2011	Retrospective	SES	68	317(63.9)	496	183	6–9 m	2.342(1.117–4.908)
Jørgensen E(S9)	2001	RCT	BMS	60	293(79.4)	369	29	6 m	3(1–8.7)
Kamitani T(S10)	2005	Prospective	BMS	61.8	97(89.0)	109	51	6 m	0.96(0.324–2.82)
Kim JS(S11)	2009	Retrospective	SES	56	394(70.7)	557	142	9 m	1.81(0.77–4.29)
Kuwano T(S12)	2011	Retrospective	B/D	67	859(79.8)	1076	540	8 m	1.32(0.87–2.01)
Liistro F(S13)	2006	Prospective	SES	66	188(77.0)	244	61	9 m	3.21(1.01–6.4)
Niroomand F(S14)	2004	Retrospective	BMS	63.5	225(100.0)	225	48	6 m	2.08(1.12–3.84)
Ribichini F(S15)	2003	Retrospective	BMS	62.5	737(82.2)	897	125	6 m	2.34(1.61–3.41)
Rathore S(S16)	2009	Retrospective	SES-PES	68.2	1413(75.0)	1885	697	9 m	1.45(1.07–1.97)
Rittersma SZH(S17)	2004	Prospective	BMS	58	278(80.6)	345	32	6–10 m	0.87(0.34–2.25)
Sahara M(S18)	2004	Retrospective	BMS	66.3	80(86.9)	92	18	6 m	2.494(0.716–8.344)
Xu YL(S19)	2011	Prospective	SES-PES	57	237(78.2)	303	86	8 m	2.046(0.933–4.485)
Zairis MN(S20)	2002	Prospective	BMS	59.3	396(82.0)	483	95	6 m	2.12(1.3–3.45)

**Table 2:** Characteristics of 18 studies investigating MACE in the meta-analysis.

Study	Year	Design	Stent	Age	Male	Total Patients	DM patients	FU	OR(95%CI)
Briguori M(S21)	2005	Retrospective	SES	65.9	781(77.2)	1012	222	5 y	1.45(0.62–3.56)
Cosgrave J(S22)	2005	Prospective	SES-PES	62.3	465(87.9)	529	127	9 m	2.06(1.37–3.10)
Fath-Ordoubadi F (S23)	2012	RCT	DES	63.1	1268(77.3)	1640	462	2 y	1.54(1.11–2.213)
Fujiwara K(S24)	2002	Retrospective	BMS	64.2	268(83.0)	323	214	6 m	1.99(1.23–3.22)
Gao RL(S25)	2008	Prospective	DES	60	967 (81.3)	1189	271	9 m	2.149(1.085–4.256)
Gurvitch R(S26)	2010	Prospective	DES	62.6	404(71.6)	564	83	12 m	1.75(0.96–3.20)
Hoffmann R(S27)	2007	Retrospective	B/D	60	595(80.1)	734	164	27.5 m	2.14(1.48–3.07)
Ijsselmuiden AJJ(S7)	2003	RCT	BMS	61	324(81.0)	400	32	6 m	2.22(1.10–4.40)
Kralev S(S28)	2009	Prospective	BMS	65	291(73.9)	394	95	6 m	1.42(0.86–2.35)
Kuchulakanti PK(S29)	2005	Prospective	SES	64.1	897(63.8)	1407	496	6 m	2.3(0.9–5.8)
Lee MS(S30)	2008	Retrospective	DES	68	651(73.4)	887	244	12 m	2.10(1.06–4.16)
Lee SR(S31)	2008	Prospective	SES	62	1123(72.8)	1541	380	6 m	1.167(0.680–2.004)
Nakamura M(S32)	2010	Prospective	SES	66.2	641(72.1)	889	889	3 y	1.535(1.034–2.279)
Novack V(S33)	2009	Prospective	DES	63.3	494(68.4)	722	256	12 m	1.00(0.71–1.39)
Ogita M(S34)	2011	Retrospective	BMS	64.7	628(63.9)	983	271	2214 d	1.03(0.65–1.64)
Patsa C(S35)	2011	Prospective	DES	61.8	423(82.7)	511	173	20 m	2.01(0.99–4.11)
Yan BP(S36)	2011	Prospective	DES	64.6	6875(74.7)	9204	2209	12 m	1.3(1.1–1.54)
Zahn R(S37)	2010	RCT	SES	64.8	8826(75.5)	10894	3197	6.4 m	1.48(1.16–1.90)

**Table 3:** Characteristics of 18 studies investigating ST in the meta-analysis.

Study	Year	Design	Stent	Age	Male	Total Patients	DM patients	FU	OR(95%CI)
Daemen J1(S38)	2007	RCT	SES-PES	62.6	6065(75%)	8146	1315	3 y	0.67(0.2–2.27)
									1.22(0.34–4.34)
Hong SJ(S39)	2010	RCT	SES-PES	65.9	125(74.0)	169	169	3 y	1.347(0.266–6.83)
Kimura T(S40)	2012	Prospective	SES	68.4	9643(75.0)	12824	6312	5 y	3.86(1.51–9.88)
Li Y(S41)	2011	Prospective	SES	64.2	757(73.6)	1029	235	12 m	6.852(2.091–22.453)
Machecourt J(S42)	2007	Prospective	SES	61.9	1291(74.5)	1731	844	3 y	2.7(1.4–5.2)
Palmerini T(S43)	2011	RCT	B/D	63.7	5260(73.4)	7162	1924	12 m	2.39(1.53–3.72)
Park DW(S44)	2009	Prospective	DES	62.7	2229(70.5)	3160	865	29 m	0.62(0.2–1.94)
Pinto Slottow TL(S45)	2008	Retrospective	DES	64.5	5492(63.4)	8402	2901	2 y	1.9(1.2–3.1)

**Table 4:** Characteristics of 8 studies investigating TLR in the meta-analysis.

Study	Year	Design	Stent	Age	Male	Total Patients	DM patients	FU	OR(95%CI)
Cosgrave J(S46)	2007	Retrospective	SES-PES	62.9	1109(86.4)	1283	344	9 m	2.34(1.5–3.65)
Freixa X(S47)	2012	Retrospective	DES	62.712	86(74.1)	116	73	20.4 m	1.18(0.51–2.72)
Hoffmann R(S27)	2007	Retrospective	B/D	60	595(80.1)	734	164	27.5 m	1.93(1.13–3.36)
Kimura T(S40)	2012	Prospective	SES	68.4	9643(75.0)	12824	6312	<1 y	1.44(1.26–1.65)
									1.19(1.04–1.37)
Nakamura M(S32)	2010	Prospective	SES	66.2	641(72.1)	889	889	3 y	1.676(1.018–2.716)
Naidu SS(S48)	2012	Prospective	ESE	64.5	5612(69.6)	8061	2860	12 m	1.6(1.23–2.07)
Sardi GL(S49)	2011	Prospective	DES	66.2	109(55.1)	198	136	12 m	1.16(0.68–1.97)
Tahara S(S50)	2011	Prospective	SES	63.7	892(66.7)	1336	385	12 m	1.3(0.65–2.6)

**Table 5:** Characteristics of 8 studies investigating TVR in the meta-analysis.

Study	Year	Design	Stent	Age	Male	Total Patients	DM patients	FU	OR(95%CI)
Agema WRP(S51)	2004	Prospective	BMS	62.1	2250(70.1)	3177	495	9.6 m	1.57(1.19–2.07)
Akin I(S52)	2010	Prospective	SES-PES	66.7	3910(74.9)	5218	1659	12 m	1.438(0.994–2.081)
Gurvitch R(S26)	2010	Prospective	DES	62.6	404(71.6)	564	83	12 m	1.45(0.66–3.18)
Lemos PA(S53)	2004	Prospective	SES	61	670(70.0)	958	160	12 m	1.81(1.1–2.99)
Marzocchi A(S54)	2007	Prospective	B/D	68	7992(75.2)	10629	2635	2 y	1.26(1.09–1.46)
Park DW(S44)	2009	Prospective	DES	62.7	2229(70.5)	3160	865	29 m	1.75(1.1–2.78)
Singh M(S55)	2005	RCT	BMS	59.9	8893(77.4)	11484	2684	9 m	1.088(0.85–1.392)
									1.53(1.22–1.92)
Zahn R(S37)	2010	RCT	SES	64.8	8826(75.5)	10894	3197	6.4 m	1.05(0.89–1.25)

### ISR

**Figure 1:**
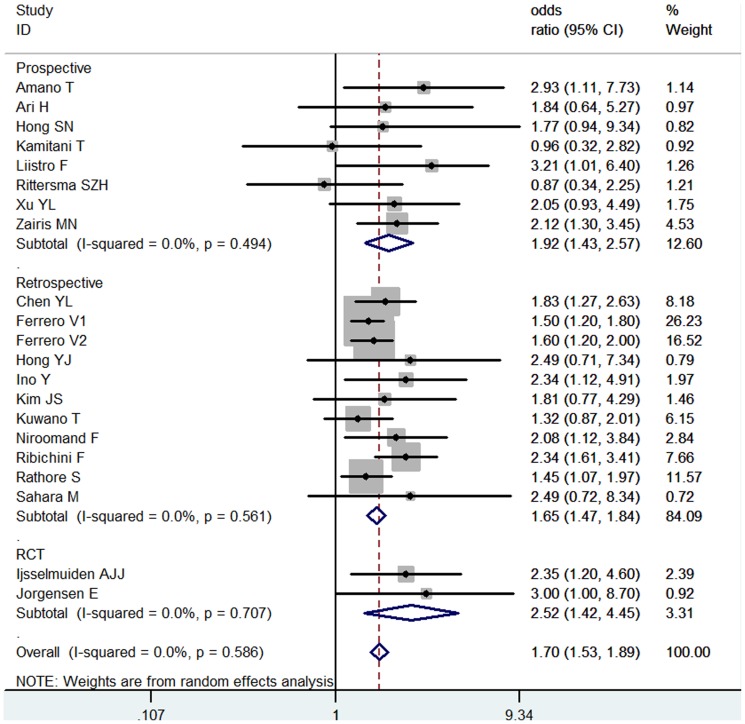
Meta- analysis of the association between DM and ISR.

21 studies including 9,578 total patients and 2,667 DM patients, investigated the association between DM and ISR after coronary stenting. 12 studies were BMS implantation, 5 were DES implantation, and 4 were mixed stent implantation. Overall, there was significant association between DM and ISR (OR = 1.70, 95% CI: 1.53–1.89) (Figure 1), no significant heterogeneity was identified (I^2^ = 0.0%, *P* = 0.586). Subgroup analysis showed that the association remained significant in BMS (OR = 1.76, 95%CI = 1.54–2.00) as well as DES patients (OR = 1.70, 95% CI: 1.33–2.17) (Figure S2) (*P* interaction = 0.972). Little publication bias was detected (Egger's test: *P* = 0.054).

### MACE

**Figure 2:**
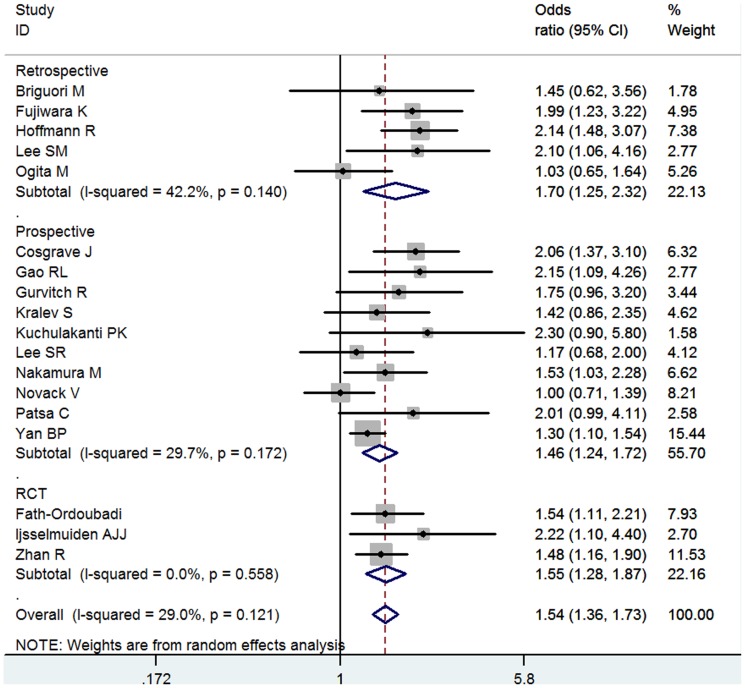
Meta- analysis of the association between DM and MACE.

18 studies, consisting 33,823 total patients and 9,785 DM patients, investigated the association between DM and MACE after coronary stenting. 13 studies were DES implantation, 4 were BMS implantation, 1 was mixed. After stenting, the overall of DM with MACE was significant (OR = 1.54, 95% CI: 1.36–1.73) (Figure 2). The heterogeneity was mild (I^2^ = 29.0%, *P* = 0.121). Subgroup analysis showed that the DM was associated with MACE in BMS (OR = 1.68, 95% CI: 1.25–2.26) as well as DES (OR = 1.47, 95% CI: 1.30–1.66) implantation (Figure S3). Interestingly, we only found the significant association in the subgroup with follow-up duration less than 3 years (OR = 1.57, 95% CI: 1.39–1.78) rather than the one with over 3 years follow-up (OR = 1.11, 95% CI: 0.74–1.67) (Figure S4). However, the interaction tests by using method from Altman et al [21] failed to identify significant difference between these 2 subgroups (*P* interaction = 0.163). In addition, the significance was similar in the subgroups whose follow-up duration was less than 3 years (Less than 1 year, OR = 1.51, 95% CI: 1.25–1.82; 1–3 years, OR = 1.66, 95% CI: 1.41–1.94). The marginal publication bias was detected (Egger's test: *P* = 0.046).

### ST

**Figure 3:**
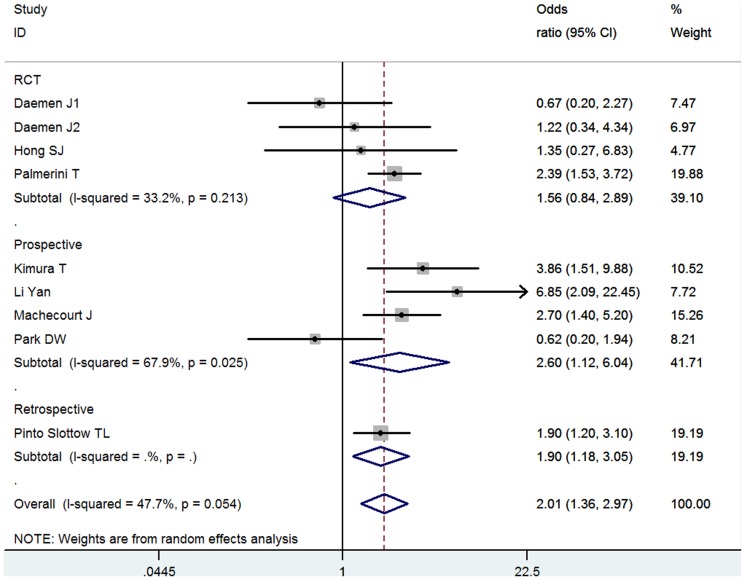
Meta- analysis of the association between DM and ST.

There were 8 eligible studies involving 42,623 total patients and 14,565 DM patients. 7 studies were DES implantation and 1 was mixed. Overall association between DM and thrombus after stenting was significant (OR = 2.01, 95% CI: 1.36–2.97) (Figure 3) and moderate heterogeneity existed (I^2^ = 47.7%, *P* = 0.054). Subgroup analysis showed there was significant association in the subgroup whose follow-up duration was less than 1 year (OR = 3.48, 95%CI = 1.30–9.37), but not in over 1 year (OR = 1.49, 95%CI = 0.93–2.38) (Figure S5). Significant difference between subgroups was not detected (*P* interaction = 0.152). No significant publication bias was indicate by Egger's test (*P* = 0.496).

### TLR

**Figure 4:**
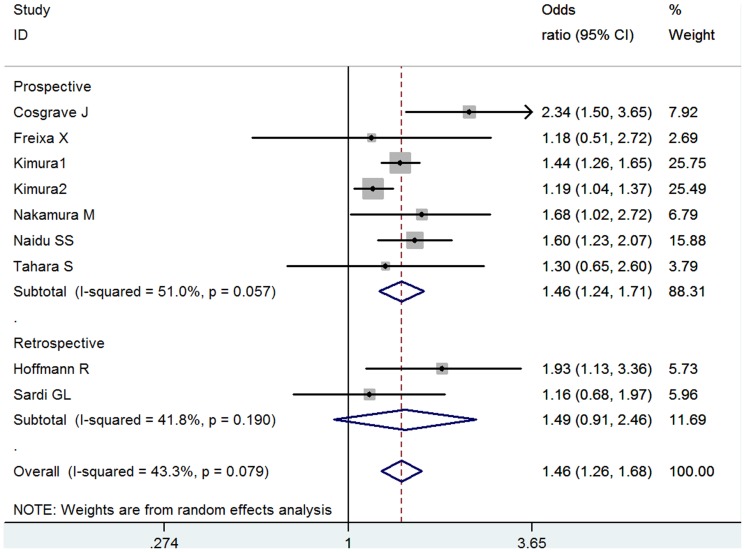
Meta- analysis of the association between DM and TLR.

8 studies including 25,441 total patients and 11,163 DM patients were eligible. Almost all the studies used DES implantation except one. Overall, there was significant association between DM and TLR (OR = 1.46, 95% CI: 1.26–1.68) (Figure 4). Moderate heterogeneity existed (I^2^ = 43.3%, *P* = 0.079). In the subgroups according to follow-up yeas, the association remained significant (Figure S5) (*P* interaction = 0.330). No significant publication bias was detected (Egger's test: *P* = 0.286).

### TVR

**Figure 5:**
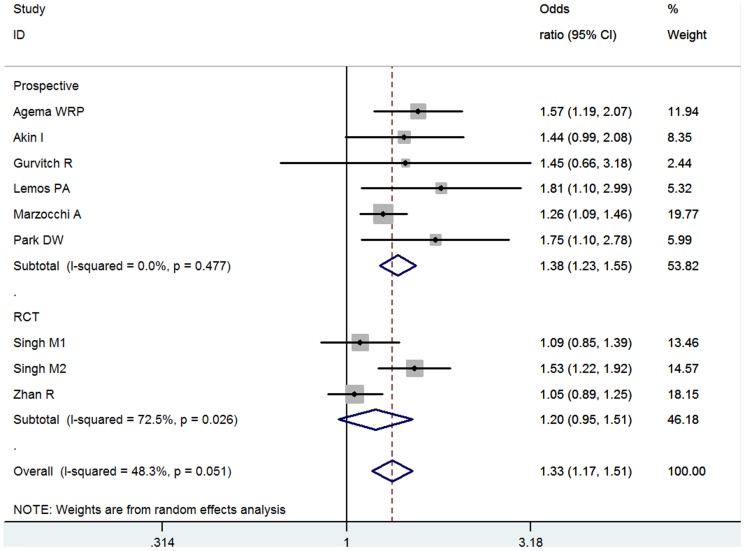
Meta- analysis of the association between DM and TVR.

8 studies involving 46,084 total patients and 11,778 DM patients were eligible. 2 studies were BMS implantation, 1 studies mixed. Overall association was significant between DM and MACE (OR = 1.33, 95% CI: 1.17–1.51) (Figure 5). Moderate heterogeneity was found (I^2^ = 48.3%, *P* = 0.051). The significance was similar in the groups according to stent type (BMS, OR = 1.38, 95% CI: 1.09–1.73; DES, OR = 1.39, 95% CI: 1.07–1.80) implantation (Figure S6) and follow-up years (Less than 1 year, OR = 1.33, 95% CI: 1.13–1.58: over 1 year, OR = 1.38, 95%CI: 1.03–1.83) (Figure S7). Egger's test indicated no significant publication bias (*P* = 0.106).

## Discussion

DM patients commonly have increased risk of CAD with more severe disease phenotypes than non-DM controls [23], [24]. Moreover, they have a larger extent of raised atherosclerotic coronary lesions than those non-DM people, which are often associated with negative coronary remodeling such as longer stenotic lesions and smaller diameter vessels [25], [26]. These unfavorable features for PCI result in less efficiency of treatment for both short and long term follow up. DES represents a breakthrough technology for PCI. In a number of randomized trials, DES is powerful and effective for revascularization and reducing restenosis [27]–[29]. Bangalore et al. recently reported the treatment effects by using DES appear to be more efficacious than BMS in DM patients [30]. However, the current opinion of the impact of DM on the clinical outcome remains speculative. In the present study, after analysis of data from 128,084 total patients, we found that DM was associated with several clinical outcomes, and the corresponding negative impact was similar between BMS and DES implantation. Our results suggested there was a remarkable negative effect of DM on coronary stent implantation. Hence, DM should also be taken into account cautiously in the treatment of patients by stent placement.

Generally, ISR, TLR and TVR, are critical indicators of efficacy of stenting to prevent restenosis. We reported there was a significant association between DM and ISR, TLR and TVR. These associations remained remarkable even after one year follow-up, regardless of stent type. Even DES implantation has great benefits in reducing occurrence of ISR, TLR and TVR compared to BMS implantation [14]–[16], the important impact of DM should be considered in the PCI.

The present study found that DM was associated with MACE without significant difference between BMS and DES subgroups. The result was similar among three registry studies [31]–[33], suggesting DM may increase occurrence of cardiac death, myocardial infarction and repeat revascularization. Although our results showed that this association was not significant after three years follow-up, only two studies were included in this subgroup analysis. The interaction test further indicated no difference between these two subgroups. Therefore, it was uncertain whether the association between DM and MACE remained after a long-term follow-up. In addition, the components of MACE were not presented or the definition of MACE was slightly different between some studies. Therefore this result should be interpreted with caution. A large prospective study with longer-term follow-up should be warranted in the future.

It is reported that the ST incidence after stent implantation ranges from 0.5% to 2%, and the ST mortality could even reach 45% [34]. Vascular response to stenting, platelets and coagulation plays important roles in the occurrence of ST [35]. Moreover, ST risk might be even greater in DM patients [5], [6]. Our results showed that after DES implantation within one year follow-up, the liability of DM patients to have ST was two times higher than those non-DM patients. However, the effect of DM was not significant in the subgroup that was followed up more than one year, suggesting this effect might alleviate in a longer-term. Hence, the further studies are warranted.

The present meta-analysis has several advantages in comparison to previous studies. First, our analysis of 55 studies with a larger sample size has a great chance to provide reliable estimates. Second, the odds ratios using in our meta-analysis were all extracted from multivariate regression analysis after adjustment, which might provide a more reliable and accurate assessment. Third, little heterogeneity was detected, suggesting the reliability of the results.

Several limitations should be considered. First, in several studies, the medical therapy to achieve recommended glycemic control targets and management of usual risk factors in these patients did not present in several included studies. No information concerning the level of glycemic control of these patients as well as their medical regimen was given, and medical regimens were simplified into insulin-requiring versus non–insulin-requiring. The validity of analysis was uncertain given these uncontrolled variables. Second, although all the included studies presented the inclusion and exclusion criteria of subjects, the percentage of each specific disease was not specifically presented. Due to a variety of incidence rates of ISR or ST among several common coronary artery diseases, it remains unclear whether our results from the mixed diseases could be used to extrapolate the real situation of a single one, such as the stable angina, unstable angina, and ST-segment-elevation myocardial infarction [36]–[38]. Third, although all the eligible studies reported that they collected consecutive patients' data, some included studies were retrospective design which might cause several potential biases such as selection bias, misclassification and information bias. Fourth, due to the limited data available, we could not be able to further analyze the effect of DM on different type of DES, such as sirolimus-eluting stent (SES) and paclitaxel-eluting stent (PES). Several studies reported that the incidence of ISR and some other post-procedural complications are different between SES and PES [39], [40]. Fifth, there was a moderate publication bias in the analysis of DM and MACE (Egger's test: *P* = 0.046), which indicates a bias of literature selection. Hence, the results should be interpreted with caution and more studies on relationship between DM and MACE are warranted in the future.

In summary, we suggest DM has a pivotal effect on a number of clinical outcomes after coronary stenting. It might play a profound role in the development of ISR, MACE, ST, TLR, and TVR. The complicated mixed effect should be considered in treatment DM patients.

## Supporting Information

Checklist S1 PRISMA 2009 Checklist.
(DOC)

Figure S1 Flow diagram of the study selection process.
(TIF)

Figure S2 Subgroup analysis of ISR according to stent type.
(TIF)

Figure S3 Subgroup analysis of MACE according to stent type.
(TIF)

Figure S4 Subgroup analysis of MACE according to follow-up.
(TIF)

Figure S5 Subgroup analysis of ST according to follow-up.
(TIF)

Figure S6 Subgroup analysis of TLR according to follow-up.
(TIF)

Figure S7 Subgroup analysis of TVR according to stent type.
(TIF)

Figure S8 Subgroup analysis of TVR according to follow up.
(TIF)

File S1 Supplementary Appendix.
(DOC)
