# Supplementary figures and images for: The Association of Diabetes Mellitus with Clinical Outcomes after Coronary Stenting: A Meta-Analysis
Source: PLoS One. 2013 Sep 16;8(9):e72710. doi: 10.1371/journal.pone.0072710 (PMC3774683; doi:10.1371/journal.pone.0072710)

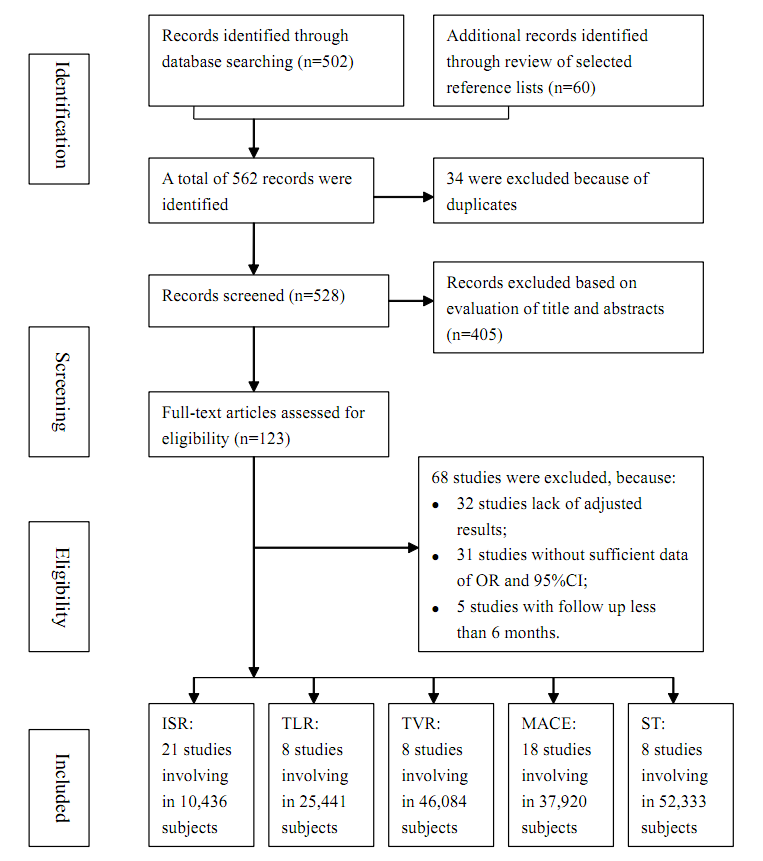

Supplement: Figure S1 — Flow diagram of the study selection process. (TIF) [file pone.0072710.s002.tif]

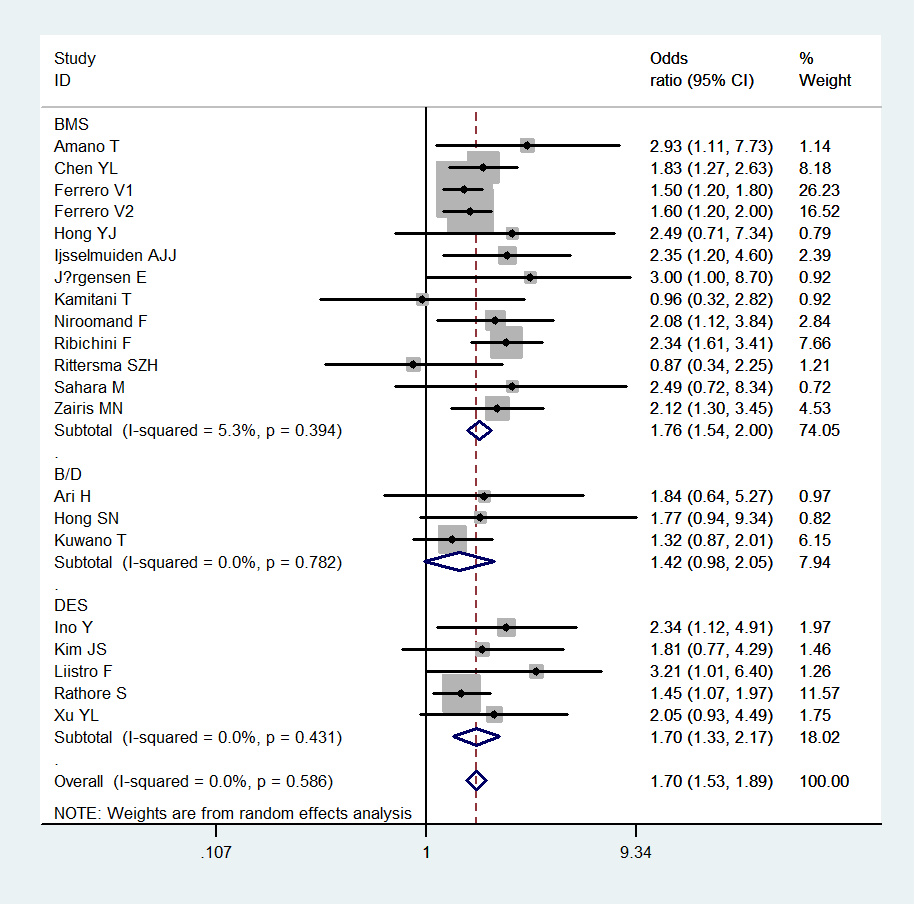

Supplement: Figure S2 — Subgroup analysis of ISR according to stent type. (TIF) [file pone.0072710.s003.tif]

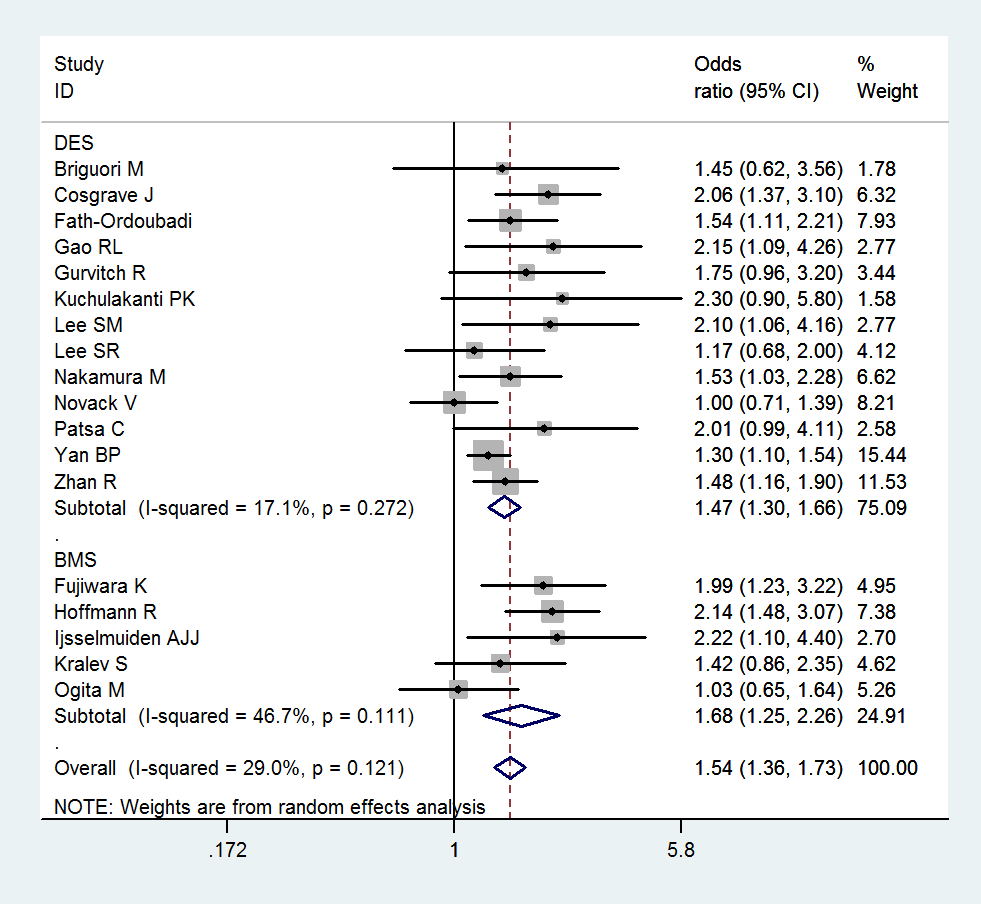

Supplement: Figure S3 — Subgroup analysis of MACE according to stent type. (TIF) [file pone.0072710.s004.tif]

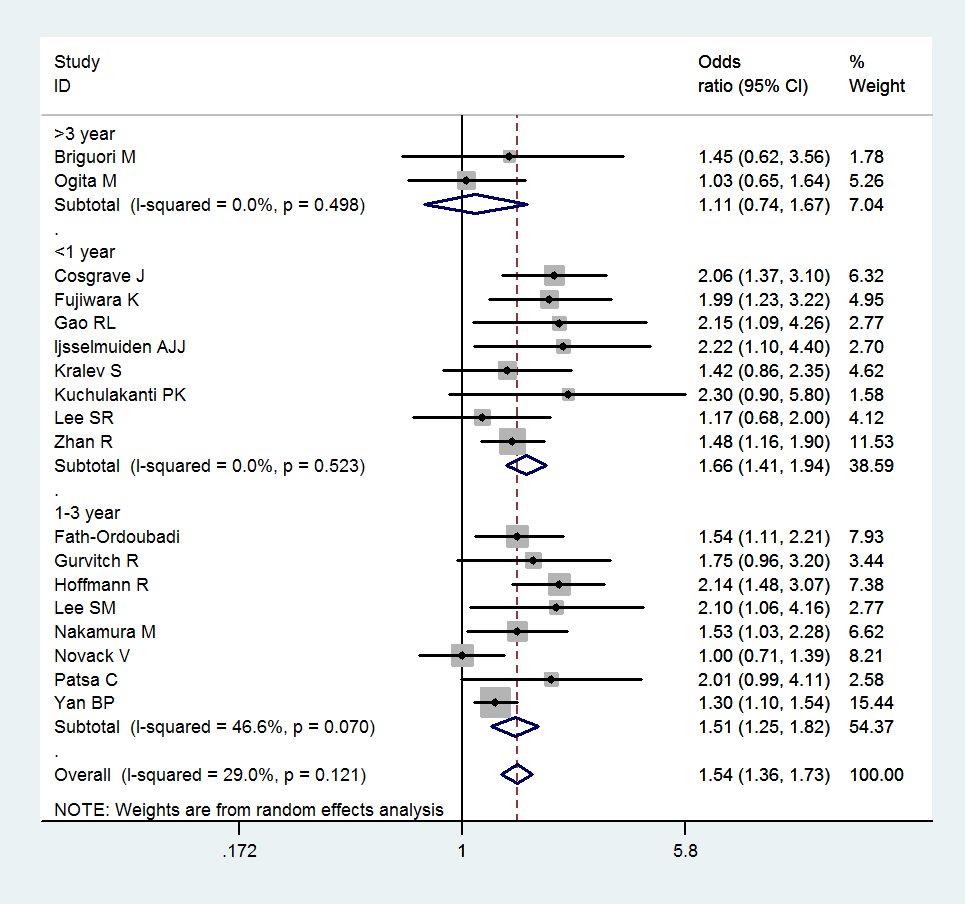

Supplement: Figure S4 — Subgroup analysis of MACE according to follow-up. (TIF) [file pone.0072710.s005.tif]

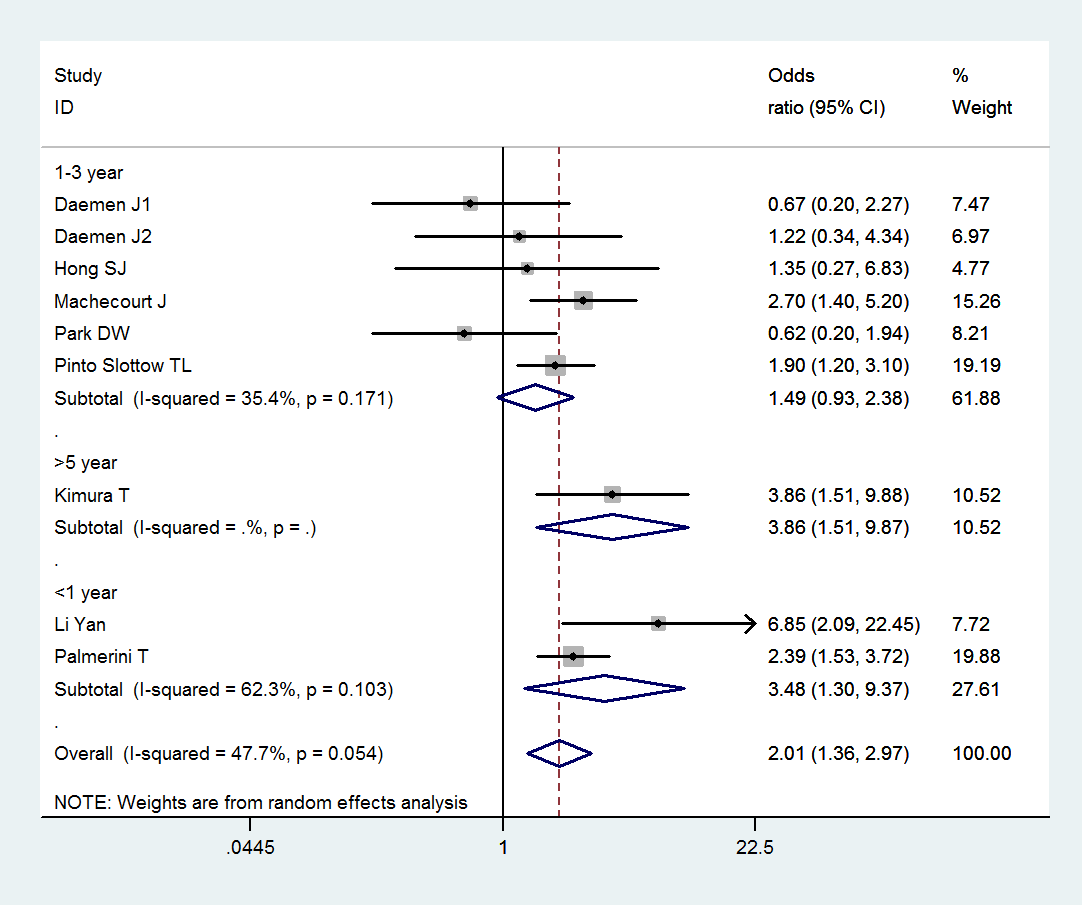

Supplement: Figure S5 — Subgroup analysis of ST according to follow-up. (TIF) [file pone.0072710.s006.tif]

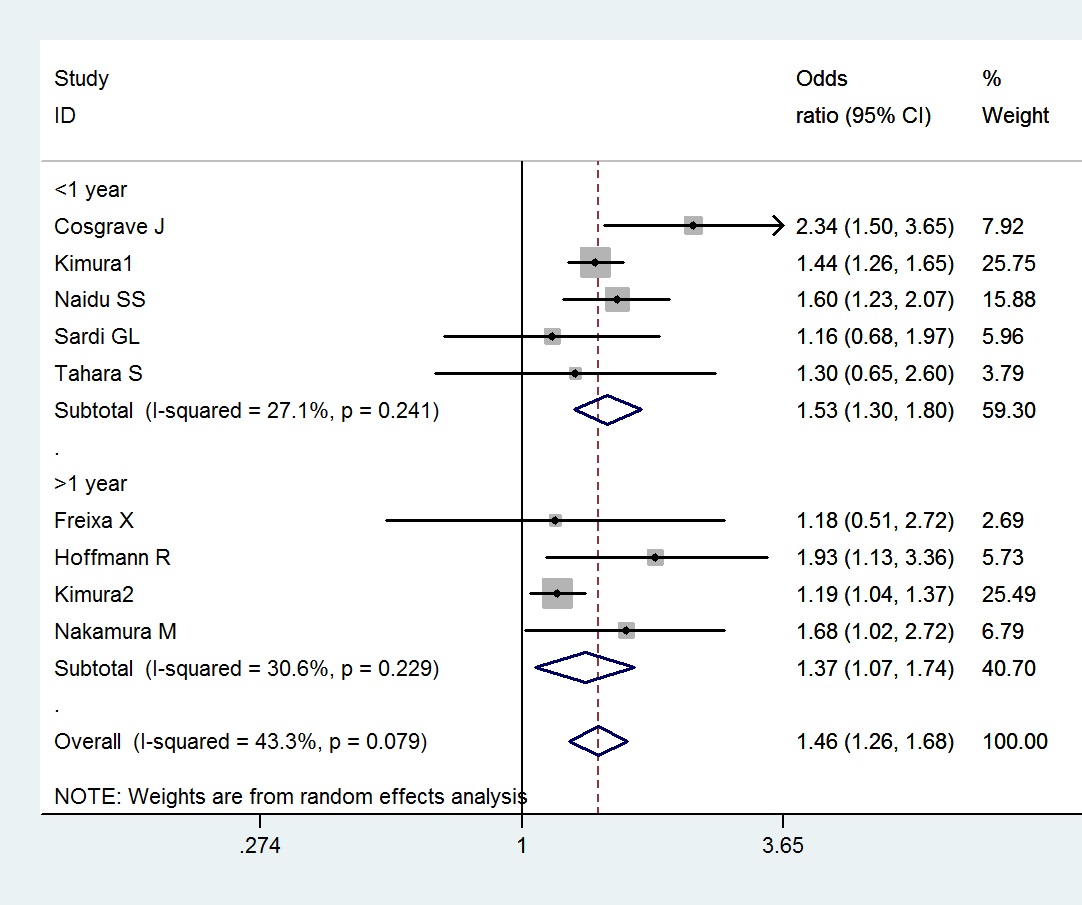

Supplement: Figure S6 — Subgroup analysis of TLR according to follow-up. (TIF) [file pone.0072710.s007.tif]

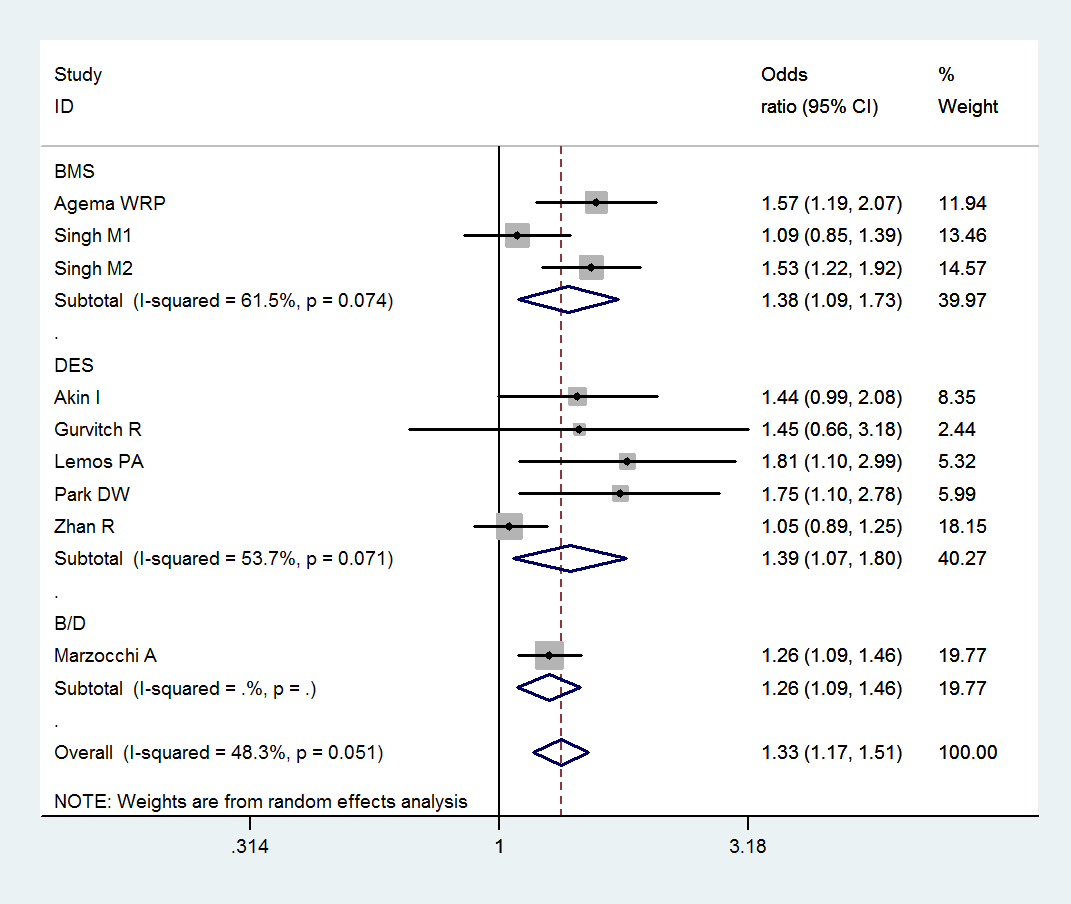

Supplement: Figure S7 — Subgroup analysis of TVR according to stent type. (TIF) [file pone.0072710.s008.tif]

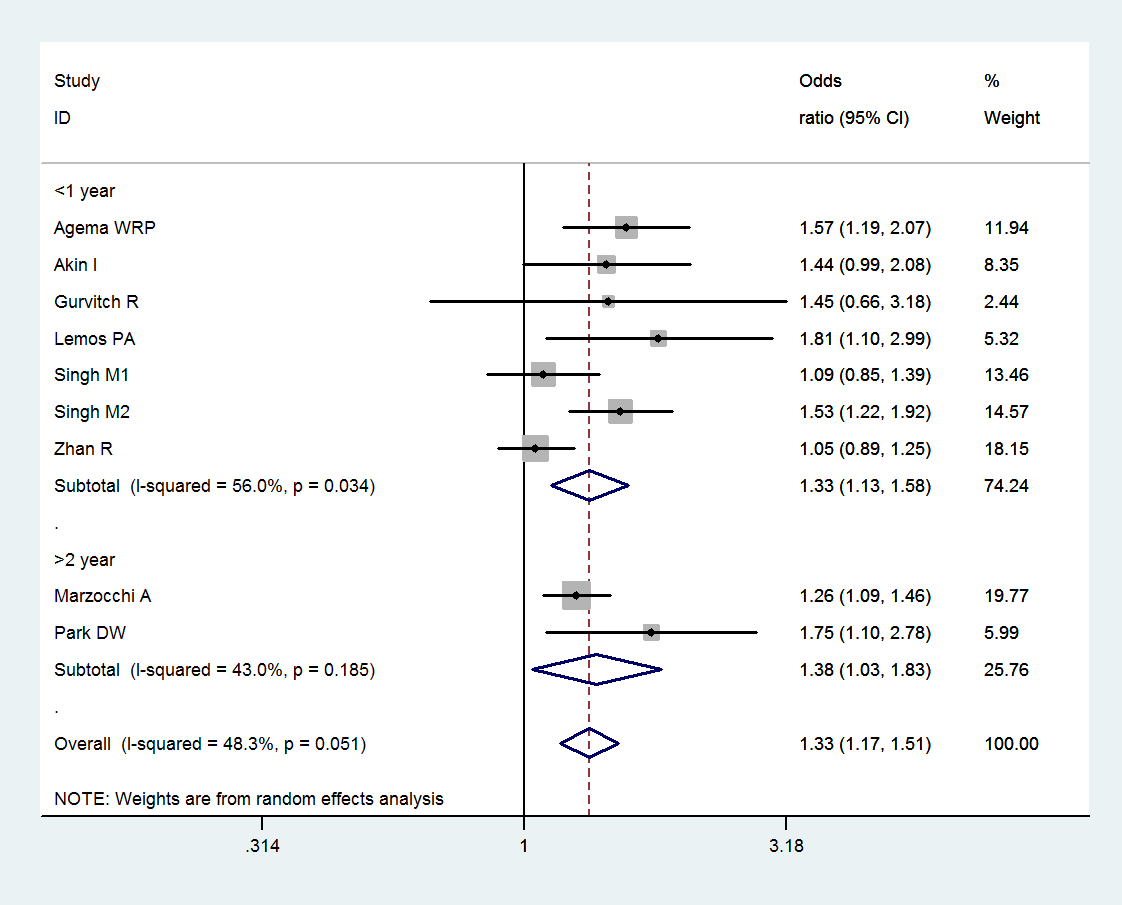

Supplement: Figure S8 — Subgroup analysis of TVR according to follow up. (TIF) [file pone.0072710.s009.tif]
