# Supplementary material for: The Association of Diabetes Mellitus with Clinical Outcomes after Coronary Stenting: A Meta-Analysis
Source: PLoS One. 2013 Sep 16;8(9):e72710. doi: 10.1371/journal.pone.0072710 (PMC3774683; doi:10.1371/journal.pone.0072710)
Supplement: File S1 — Supplementary Appendix. (DOC) [file pone.0072710.s010.doc]

**Supplementary Appendix**

**The association of diabetes mellitus with clinical outcomes after coronary stenting: a meta-analysis**

Shan-Yu Qin, Hai-Xing Jiang*, Bang-Li Hu, You Zhou, Ming-Zhi Xie, Lin Tao

*To whom correspondence should be addressed. E-mail: 1391510812@qq.com

This supplement included: Table S1-S7, Reference S1-S55, and Figure S1-S7

Table S1 Characteristics of 20 studies investigating ISR in the meta-analysis

| Study | Duration | DM diagnosis | DM Treatment | Disease | Registry name | Adjusted confounding factors |
| --- | --- | --- | --- | --- | --- | --- |
| Amano T | Aug.2003-Oct.2004 | NA | NA | SP | No | ⑧⑨Aldosterone |
| Ari H | Sep.2004-Apr.2006 | NA | NA | CAD | No | ①-⑨⑫ADMA |
| Chen YL | Jan.1998-Dec.2000 | NA | NA | Stable CAD | No | ⑧⑨ |
| Ferrero V | 1993-2000 | non–insulin  dependent | NA | SP, USP, MI, | No | ①-⑥, ⑧⑨ |
| Hong SN | Jan.2003-Dec.2003 | NA | NA | SA, USP, MI,  ST-or NSTEACS | No | ④⑧⑨CRP |
| Hong YJ | Nov.2003-Jun.2005 | NA | NA | SP, USP, MI | No | ①-⑨BNP |
| Ijsselmuiden AJJ | Jan.1999-Jun.2001 | NA | NA | SP, USP, MI | No | ①-⑦ CRP |
| Ino Y | Sep.2004-Aug.2007 | NA | NA | SP, USP, MI,  ACS | No | ①-⑥, ⑧⑨⑩  Hinge motion |
| Jørgensen E | Apr.1997-Jan.2000 | NA | NA | USP, MI | DANSTENT | ①-⑨ |
| Kamitani T | Jan.1996-Dec.2001 | WHO criteria  or medications | NA | CAD | No | ①-⑥, ⑧⑨Lp(a) |
| Kim JS | Dec.2003-Feb.2006 | NA | NA | CAD, ACS | No | ①-⑨stent fracture |
| Kuwano T | Mar.2003-May.2009 | NA | NA | CAD, except MI | FU registry | ①-⑩total bilirubin |
| Liistro F | Jul.2002-Mar.2005 | NA | NA | SA, USP, MI,  NSTEACS | TRUE | ①-⑥, ⑧⑨ |
| Niroomand F | NA | NA | NA | CAD, except MI | No | ①-⑨ |
| Ribichini F | 1993-2000 | NA | NA | SP, USP, MI,  silent ischemia | No | ①-⑦ |
| Rathore S | May.2004-Dec.2007 | NA | NA | SP, USP, MI | No | ①-⑨ |
| Rittersma SZH | NA | NA | NA | SP, USP, MI | No | ①-⑨CRP |
| Sahara M | Jan.2000-Jun.2001 | medications | NA | CAD | No | ④⑧⑨Soft plaque |
| Xu YL | Apr.2007-Mar.2009 | NA | NA | CAD, except MI | No | ①-⑨CRP |
| Zairis MN | Jan.1998-Jun.2000 | NA | NA | SA, USP, MI,  ST-or NSTEACS | GENERATION | ①-⑨CRP Lp(a) |

①age, ②gender, ③smoking, ④hypertension, ⑤body mass index, ⑥serum lipid, ⑦drug: Nitrate, angiotensin-converting enzyme, angiotensin II, Statin, calcium antagonists, ⑧vessel: lesion narrowness, length of lesion, minimal lumen diameter, reference diameter; type of lesion; ⑨number of stents,

stent length/lesion; stent diameter, stent type; ⑩renal insufficiency, glomerular filtration rate; ⑪LVF: left ventricular function ⑫CAD, stable-unstable angina, ST or non ST-segment elevated myocardial infarction

Table S2 Characteristics of 18 studies investigating MACE in the meta-analysis

| Study | Duration | DM diagnosis | Treatment | Disease | Registry name | Adjusted confounding factors |
| --- | --- | --- | --- | --- | --- | --- |
| Briguori M | Apr.2003-May.2004 | Insulin treatment | Insulin or oral drugs | SP, USP,  silent ischemia | No | ⑩clopidogrel discontinuation  Complete revascularization |
| Cosgrave J | Mar.2003-Feb.2004 | Dietary therapy,  Insulin or oral drugs | Dietary therapy,  Insulin or oral drugs | SP, USP | No | ①-⑥intra-aortic  balloon pump use |
| Fath-Ordoubadi F | 2008-2009 | Dietary therapy,  Insulin or oral drugs | Dietary therapy,  Insulin or oral drugs | ACS | NOBORI-2 | ①-④ |
| Fujiwara K | Jan.1998-Jun.2000 | WHO, insulin  or oral drugs | NA | AMI | No | ①-⑧ |
| Gao RL | Ju.2004-Nov.2004 | NA | NA | CAD except MI  Within 7days | No | ③④⑧⑨bifurcation, occlusion,  post-procedure TIMI flow |
| Gurvitch R | Aug.2004-May.2006 | NA | NA | STEMI | No | ①-④⑧⑩  Cerebrovascular disease |
| Hoffmann R | Jun.2003-jun.2005 | NA | NA | SP, USP | No | ①-⑧metabolic syndrome |
| Ijsselmuiden AJJ | Jan.1999-Jun.2001 | NA | NA | SP, USP, MI, | No | ①-⑩CRP  Left anterior descending |
| Kralev S | 2004–2006 | WHO | NA | STEMI | No | ①-⑥creatine kinase, glycoprotein  IIb/IIIa receptor inhibitor |
| Kuchulakanti PK | Apr.2003-Aug.2004 | Insulin treatment | Insulin and non  Insulin dependent | SA, USP, MI | C-REWARDS | ①-⑧ |
| Lee MS | NA | NA | NA | ACS | No | ①-⑨⑫Ejection fraction |
| Lee SR | Nov.2005-Sep.2006 | NA | NA | AMI | No | ②⑧⑨left coronary artery |
| Nakamura M | Sep.2004.Sep.2005 | HbA 1c, insulin  or oral drugs | Insulin-treated | SP, USP, MI,  silent ischemia | Cypher J-PMS | ③⑥⑧Hemodialysis  Ejection fraction |
| Novack V | Jul.2004-Jun.2006 | insulin  or oral drugs | insulin  or oral drugs | SP, USP, MI | EVENT | ①-⑩ |
| Ogita M | Jan.1999-Aug.2004 | HbA 1c, insulin  or oral drugs | NA | SP, USP, MI,  ACS | No | ①③⑧⑩HbA1c  Insulin usage |
| Patsa C | Mar.2006-Oct.2009 | WHO | insulin  or oral drugs | SP, silent ischemia | No | ①-⑧metabolic syndrome |
| Yan BP | Apr.2004-Mar.2008 | NA | NA | USP, MI,  STEMI, NSTEMI | MIG | ①-⑧cardiogenic shock  Cerebrovascular disease |
| Zahn R | Apr.2002-Sep.2005 | NA | NA | SP, USP,  STEMI, NSTEMI | German Cypher  Stent Registry. | ①-⑩  left ventricular function |

①age, ②gender, ③smoking, ④hypertension, ⑤body mass index, ⑥serum lipid, ⑦drug: Nitrate, angiotensin-converting enzyme, angiotensin II, Statin, calcium antagonists, ⑧vessel: lesion narrowness, length of lesion, minimal lumen diameter, reference diameter; type of lesion; ⑨number of stents, stent length/lesion; stent diameter, stent type; ⑩renal insufficiency, glomerular filtration rate; ⑪LVF: left ventricular function ⑫CAD, stable-unstable angina, ST or non ST-segment elevated myocardial infarction;

Table S3 Characteristics of 8 studies investigating ST in the meta-analysis

| Study | Duration | DM diagnosis | Treatment | Disease | Registry name | Adjusted confounding factors |
| --- | --- | --- | --- | --- | --- | --- |
| Daemen J1 | Apr.2002-Dec.2005 | NA | NA | SP, USP, MI,  ACS | T-SEARCH | ①-⑪ |
| Hong SJ | Apr.2005-Jan.2006 | WHO, insulin  or oral drugs | Dietary therapy,  Insulin or oral drugs | SP, USP,  silent ischemia | No | ①-⑨ |
| Kimura T | Aug.2004-Nov.2006 | WHO, insulin  or oral drugs | insulin  or oral drugs | ACS | j-Cypher registry | ①-⑪ |
| Li Y | Sep.2006-Jul.2007 | NA | NA | SP, USP,  silent ischemia | FIREMAN | ①-⑩ |
| Machecourt J | NA | medical therapy | insulin  or oral drugs | Stable CAD,  ACS | EVASTENT | ⑧-⑪  Calcified lesion |
| Palmerini T | NA | WHO, insulin  or oral drugs | Dietary therapy,  Insulin or oral drugs | NSTE-ACS | ACUITY | ①-⑩ |
| Park DW | Feb.2003-Feb.2006 | insulin  or oral drugs | insulin  or oral drugs | SP, USP, MI | No | ①-⑪ stent type |
| Pinto Slottow TL | Apr.2003-Jun.2007 | NA | NA | CAD | No | ①-⑪  Cardiogenic shock |

①age, ②gender, ③smoking, ④hypertension, ⑤body mass index, ⑥serum lipid, ⑦drug: Nitrate, angiotensin-converting enzyme, angiotensin II, Statin, calcium antagonists, ⑧vessel: lesion narrowness, length of lesion, minimal lumen diameter, reference diameter; type of lesion; ⑨number of stents, stent length/lesion; stent diameter, stent type; ⑩renal insufficiency, glomerular filtration rate; ⑪LVF: left ventricular function

Table S4 Characteristics of 8 studies investigating TLR in the meta-analysis

| Study | Duration | DM diagnosis | Treatment | Disease | Registry name | Adjusted confounding factors |
| --- | --- | --- | --- | --- | --- | --- |
| Cosgrave J | Mar.2003-Mar.2005 | Dietary therapy,  Insulin or oral drugs | Dietary therapy,  Insulin or oral drugs | SP, USP | No | ①-⑥intra-aortic  balloon pump use |
| Freixa X | Jan.2004-Jan.2009 | NA | NA | ACS, SP,  STEMI | No | Previous BMS, Focal ISR  Stent fracture, Same-stent strategy |
| Hoffmann R | Jun.2003-Jun.2005 | NA | NA | SP, USP | No | ①-⑧metabolic syndrome |
| Kimura T | Aug.2004-Nov.2006 | WHO, insulin  or oral drugs | insulin  or oral drugs | ACS | j-Cypher registry | ①-⑪ |
| Nakamura M | Sep.2004.Sep.2005 | insulin  or oral drugs | Dietary therapy,  Insulin or oral drugs | SP, USP, MI | Cypher J-PMS | ②⑧-⑩Ostial lesion,  Bifurcation, Hemodialysis |
| Naidu SS | Jul.2008-Feb.2010 | insulin  or oral drugs | insulin  or oral drugs | SP, USP, MI | XIENCE V | ②⑧-⑩Ostial lesion |
| Sardi GL | Jan.2003-Dec.2009 | NA | NA | SP, USP | No | ①heart failure, hematocrit,  Hemodialysis |
| Tahara S | NA | HbA1c≥6.5% | Dietary therapy,  Insulin or oral drugs | SP, USP, MI | STLLR | ①-⑧Bifurcation,  Geographical miss |

①age, ②gender, ③smoking, ④hypertension, ⑤body mass index, ⑥serum lipid, ⑦drug: Nitrate, angiotensin-converting enzyme, angiotensin II, Statin, calcium antagonists, ⑧vessel: lesion narrowness, length of lesion, minimal lumen diameter, reference diameter; type of lesion; ⑨number of stents,

stent length/lesion; stent diameter, stent type; ⑩renal insufficiency, glomerular filtration rate; ⑪LVF: left ventricular function ⑫CAD, stable-unstable angina, ST or non ST-segment elevated myocardial infarction

Table S5 Characteristics of 8 studies investigating TVR in the meta-analysis

| Study | Duration | DM diagnosis | Treatment | Disease | Registry name | Adjusted confounding factors |
| --- | --- | --- | --- | --- | --- | --- |
| Agema WRP | Mar.1999-Jun.2001 | NA | NA | Stable angina | GENDER | ①-⑨Multi-vessel PTCA  Previous PTCA |
| Akin I | Oct.2005-Oct.2006 | WHO, insulin  or oral drugs | Dietary therapy,  Insulin or oral drugs | ACS | DES.DE | ⑨⑩⑫ |
| Gurvitch R | Aug.2004-May.2006 | NA | NA | STEMI | MIG | ⑨using DES  Propensity score |
| Lemos PA | Apr.2002-Apr.2003 | insulin  or oral drugs | insulin  or oral drugs | SP, USP, MI,  Cardiogenic shock | RESEARCH | ①-⑨⑫ Ostial lesion,  Bifurcation, |
| Marzocchi A | Jul.2002-Jun.2005 | NA | NA | SP, USP,  NSTEMI | REAL registry | ⑨ISR, Ostial lesion  Prior MI, PCI, CABG, |
| Park DW | Feb.2003-Feb.2006 | insulin  or oral drugs | insulin  or oral drugs | SP, USP, MI | No | ①-⑪ stent type |
| Singh M | Apr.1999-Jul.2000 | oral agent or insulin | oral agent or insulin | SP, USP | PRESTO | ①-⑨ISR, Ostial lesion  Rotablator |
| Zahn R | Apr.2002-Sep.2005 | NA | NA | SP, USP,  STEMI, NSTEMI | German Cypher  Stent Registry | ①-⑩  left ventricular function |

①age, ②gender, ③smoking, ④hypertension, ⑤body mass index, ⑥serum lipid, ⑦drug: Nitrate, angiotensin-converting enzyme, angiotensin II, Statin, calcium antagonists, ⑧vessel: lesion narrowness, length of lesion, minimal lumen diameter, reference diameter; type of lesion; ⑨number of stents,

stent length/lesion; stent diameter, stent type; ⑩renal insufficiency, glomerular filtration rate; ⑪LVF: left ventricular function ⑫CAD, stable-unstable angina, ST or non ST-segment elevated myocardial infarction

Table S6 Quality assessment for prospective and retrospective studies

| study | Clear definition of study population? | Clear definition of outcomes and outcome assessment? | Independent assessment of outcome parameters? | Sufficient duration of follow-up? | No selective loss during follow-up? | Important confounders and prognostic factors identified? |
| --- | --- | --- | --- | --- | --- | --- |
| Amano T | Yes | Yes | Yes | Yes | Yes | Yes |
| Ari H | Yes | Yes | NA | Yes | Yes | Yes |
| Chen YL | Yes | Yes | Yes | Yes | Yes | Yes |
| Ferrero V | Yes | Yes | NA | Yes | Yes | Yes |
| Hong YJ | Yes | Yes | NA | Yes | Yes | Yes |
| Hong SN | Yes | Yes | Yes | Yes | Yes | Yes |
| Ino Y | Yes | Yes | Yes | Yes | Yes | Yes |
| Kamitani T | Yes | Yes | NA | Yes | Yes | Yes |
| Kim JS | Yes | Yes | NA | Yes | Yes | Yes |
| Kuwano T | Yes | Yes | NA | Yes | Yes | Yes |
| Liistro F | Yes | Yes | Yes | Yes | Yes | Yes |
| Niroomand F | Yes | Yes | NA | Yes | Yes | Yes |
| Ribichini F | Yes | Yes | NA | Yes | Yes | Yes |
| Rathore S | Yes | Yes | NA | Yes | Yes | Yes |
| Rittersma SZH | Yes | Yes | Yes | Yes | Yes | Yes |
| Sahara M | Yes | Yes | Yes | Yes | Yes | Yes |
| Xu YL | Yes | Yes | Yes | Yes | Yes | Yes |
| Zairis MN | Yes | Yes | Yes | Yes | Yes | Yes |
| Briguori M | Yes | Yes | NA | Yes | Yes | Yes |
| Cosgrave J | Yes | Yes | NA | Yes | Yes | Yes |
| Fujiwara K | Yes | Yes | Yes | Yes | Yes | Yes |
| Gao RL | Yes | Yes | NA | Yes | Yes | Yes |
| Gurvitch R | Yes | Yes | Yes | Yes | Yes | Yes |
| Hoffmann R | Yes | Yes | Yes | Yes | Yes | Yes |
| Kralev S | Yes | Yes | Yes | Yes | Yes | Yes |
| Kuchulakanti PK | Yes | Yes | Yes | Yes | Yes | Yes |
| Lee SM | Yes | Yes | Yes | Yes | Yes | Yes |
| Lee SR | Yes | Yes | NA | Yes | Yes | Yes |
| Nakamura M | Yes | Yes | NA | Yes | Yes | Yes |
| Novack V | Yes | Yes | Yes | Yes | Yes | Yes |
| Ogita M | Yes | Yes | NA | Yes | Yes | Yes |
| Patsa C | Yes | Yes | NA | Yes | Yes | Yes |
| Yan BP | Yes | Yes | Yes | Yes | Yes | Yes |
| Kimura T | Yes | Yes | Yes | Yes | Yes | Yes |
| Li Y | Yes | Yes | Yes | Yes | Yes | Yes |
| Machecourt J | Yes | Yes | Yes | Yes | Yes | Yes |
| Park DW | Yes | Yes | Yes | Yes | Yes | Yes |
| Pinto Slottow TL | Yes | Yes | Yes | Yes | Yes | Yes |
| Cosgrave J | Yes | Yes | NA | Yes | Yes | Yes |
| Freixa X | Yes | Yes | Yes | Yes | Yes | Yes |
| Hoffmann R | Yes | Yes | Yes | Yes | Yes | Yes |
| Kimura T | Yes | Yes | Yes | Yes | Yes | Yes |
| Nakamura M | Yes | Yes | Yes | Yes | Yes | Yes |
| Naidu SS | Yes | Yes | Yes | Yes | Yes | Yes |
| Sardi GL | Yes | Yes | Yes | Yes | Yes | Yes |
| Tahara S | Yes | Yes | Yes | Yes | Yes | Yes |
| Akin I | Yes | Yes | Yes | Yes | Yes | Yes |
| Gurvitch R | Yes | Yes | Yes | Yes | Yes | Yes |
| Lemos PA | Yes | Yes | Yes | Yes | Yes | Yes |
| Marzocchi A | Yes | Yes | Yes | Yes | Yes | Yes |
| Park DW | Yes | Yes | Yes | Yes | Yes | Yes |

Table S7 Quality assessment for RCT studies

| Study | Selection bias | Performance bias | Attrition bias | Detection bias |
| --- | --- | --- | --- | --- |
| Jørgensen E | A | A | A | A |
| Fath-Ordoubadi | A | A | A | A |
| Ijsselmuiden AJJ | A | A | A | A |
| Zahn R | A | A | A | A |
| Daemen J1 | A | A | A | A |
| Hong SJ | A | A | A | A |
| Palmerini T | A | A | A | A |
| Singh M | A | A | A | A |

**References**

1. Amano T, Matsubara T, Izawa H, et al. Impact of plasma aldosterone levels for prediction of in-stent restenosis. Am J Cardiol. 2006;97:785-8.

2. Ari H, Ari S, Erdogan E, et al. A novel predictor of restenosis and adverse cardiac events: asymmetric dimethylarginine. Heart Vessels. 2010;25:19-26.

3. Chen YL, Chen MC, Wu CJ, et al. Impact of 6-month angiographic restenosis inside bare-metal stents on long-term clinical outcome in patients with coronary artery disease. Int Heart J. 2007;48:443-54.

4. Ferrero V, Ribichini F, Matullo G, et al. Estrogen receptor-alpha polymorphisms and angiographic outcome after coronary artery stenting. Arterioscler Thromb Vasc Biol. 2003;23:2223-8.

5. Hong YJ, Jeong MH, Lim SY, et al. Relation of soft plaque and elevated preprocedural high-sensitivity C-reactive protein levels to incidence of in-stent restenosis after successful coronary artery stenting. Am J Cardiol. 2006;98:341-5.

6. Hong SN, Ahn Y, Yoon NS, et al. Usefulness of serum N-terminal pro-brain natriuretic peptide to predict in-stent restenosis in patients with preserved left ventricular function and normal troponin I levels. Am J Cardiol. 2007;99:1051-4.

7. AJ IJ, Serruys PW, Scholte A, et al. Direct coronary stent implantation does not reduce the incidence of in-stent restenosis or major adverse cardiac events: six month results of a randomized trial. Eur Heart J. 2003;24:421-9.

8. Ino Y, Kubo T, Kitabata H, et al. Impact of hinge motion on in-stent restenosis after sirolimus-eluting stent implantation. Circ J. 2011;75:1878-84.

9. Jorgensen E, Kelbaek H, Helqvist S, et al. Predictors of coronary in-stent restenosis: importance of angiotensin-converting enzyme gene polymorphism and treatment with angiotensin-converting enzyme inhibitors. J Am Coll Cardiol. 2001;38:1434-9.

10. Kamitani T, Taniguchi T, Miyai N, et al. Association between plasma lipoprotein(a) concentration and restenosis after stent implantation. Circ J. 2005;69:644-9.

11. Kim JS, Lee SY, Lee JM, et al. Significant association of coronary stent fracture with in-stent restenosis in sirolimus-eluting stents. Coron Artery Dis. 2009;20:59-63.

12. Kuwano T, Miura S, Shirai K, et al. Serum levels of bilirubin as an independent predictor of coronary in-stent restenosis: a new look at an old molecule. J Atheroscler Thromb. 2011;18:574-83.

13. Liistro F, Fineschi M, Angioli P, et al. Effectiveness and safety of sirolimus stent implantation for coronary in-stent restenosis: the TRUE (Tuscany Registry of Sirolimus for Unselected In-Stent Restenosis) Registry. J Am Coll Cardiol. 2006;48:270-5.

14. Niroomand F, Hauer O, Tiefenbacher CP, et al. Influence of alcohol consumption on restenosis rate after percutaneous transluminal coronary angioplasty and stent implantation. Heart. 2004;90:1189-93.

15. Ribichini F, Wijns W, Ferrero V, et al. Effect of angiotensin-converting enzyme inhibition on restenosis after coronary stenting. Am J Cardiol. 2003;91:154-8.

16. Rathore S, Terashima M, Katoh O, et al. Predictors of angiographic restenosis after drug eluting stents in the coronary arteries: contemporary practice in real world patients. EuroIntervention. 2009;5:349-54.

17. Rittersma SZ, de Winter RJ, Koch KT, et al. Preprocedural C-reactive protein is not associated with angiographic restenosis or target lesion revascularization after coronary artery stent placement. Clin Chem. 2004;50:1589-96.

18. Sahara M, Kirigaya H, Oikawa Y, et al. Soft plaque detected on intravascular ultrasound is the strongest predictor of in-stent restenosis: an intravascular ultrasound study. Eur Heart J. 2004;25:2026-33.

19. Xu YL, Li JJ, Xu B, et al. Role of plasma C-reactive protein in predicting in-stent restenosis in patients with stable angina after coronary stenting. Chin Med J (Engl). 2011;124:845-50.

20. Zairis MN, Ambrose JA, Manousakis SJ, et al. The impact of plasma levels of C-reactive protein, lipoprotein (a) and homocysteine on the long-term prognosis after successful coronary stenting: The Global Evaluation of New Events and Restenosis After Stent Implantation Study. J Am Coll Cardiol. 2002;40:1375-82.

21. Briguori C, Colombo A, Airoldi F, et al. Sirolimus-eluting stent implantation in diabetic patients with multivessel coronary artery disease. Am Heart J. 2005;150:807-13.

22. Cosgrave J, Agostoni P, Ge L, et al. Clinical outcome following aleatory implantation of paclitaxel-eluting or sirolimus-eluting stents in complex coronary lesions. Am J Cardiol. 2005;96:1663-8.

23. Fath-Ordoubadi F, Barac Y, Abergel E, et al. Gender Impact on Prognosis of Acute Coronary Syndrome Patients Treated With Drug-Eluting Stents. Am J Cardiol. 2012.

24. Fujiwara K, Hiasa Y, Takahashi T, et al. Influence of diabetes mellitus on outcome in the era of primary stenting for acute myocardial infarction. Circ J. 2002;66:800-4.

25. Gao RL, Xu B, Lu SZ, et al. Safety and efficacy of the CYPHER Select Sirolimus-eluting stent in the "Real World"--clinical and angiographic results from the China CYPHER Select registry. Int J Cardiol. 2008;125:339-46.

26. Gurvitch R, Lefkovits J, Warren RJ, et al. Clinical outcomes of drug-eluting stent use in patients with ST elevation myocardial infarction. Int J Cardiol. 2010;143:283-8.

27. Hoffmann R, Stellbrink E, Schroder J, et al. Impact of the metabolic syndrome on angiographic and clinical events after coronary intervention using bare-metal or sirolimus-eluting stents. Am J Cardiol. 2007;100:1347-52.

28. Kralev S, Krause B, Papavassiliu T, et al. Clinical outcome of patients with diabetes presenting with ST-elevation myocardial infarction and treated with concomitant use of glycoprotein IIb/IIIa inhibitors. Cardiol J. 2009;16:234-40.

29. Kuchulakanti PK, Torguson R, Canos D, et al. Impact of treatment of coronary artery disease with sirolimus-eluting stents on outcomes of diabetic and nondiabetic patients. Am J Cardiol. 2005;96:1100-6.

30. Lee MS, Jurewitz D, Zimmer R, et al. Impact of diabetes and acute coronary syndrome on survival in patients treated with drug-eluting stents. Catheter Cardiovasc Interv. 2008;72:909-14.

31. Lee SR, Jeong MH, Ahn YK, et al. Clinical safety of drug-eluting stents in the Korea acute myocardial infarction registry. Circ J. 2008;72:392-8.

32. Nakamura M, Yokoi H, Hamazaki Y, et al. Impact of insulin-treated diabetes and hemodialysis on long-term clinical outcomes following sirolimus-eluting stent deployment. Insights from a sub-study of the Cypher Stent Japan Post-Marketing Surveillance(Cypher J-PMS) Registry. Circ J. 2010;74:2592-7.

33. Novack V, Tsyvine D, Cohen DJ, Pencina M, Dubin J, Dehghani H, et al. Multivessel drug-eluting stenting and impact of diabetes mellitus--a report from the EVENT registry. Catheter Cardiovasc Interv. 2009;73:874-80.

34. Ogita M, Miyauchi K, Kurata T, et al. Clinical impact of angiographic restenosis after bare-metal stent implantation on long-term outcomes in patients with coronary artery disease. Circ J. 2011;75:2566-72.

35. Patsa C, Toutouzas K, Tsiamis E, et al. Impact of metabolic syndrome on clinical outcomes after new generation drug-eluting stent implantation: The 'obesity paradox' phenomenon is still apparent. Nutr Metab Cardiovasc Dis. 2011.

36. Yan BP, Ajani AE, Clark DJ, et al. Recent trends in Australian percutaneous coronary intervention practice: insights from the Melbourne Interventional Group registry. Med J Aust. 2011;195:122-7.

37. Zahn R, Hamm CW, Schneider S, et al. Coronary stenting with the sirolimus-eluting stent in clinical practice: final results from the prospective multicenter German Cypher Stent Registry. J Interv Cardiol. 2010;23:18-25.

38. Daemen J, Wenaweser P, Tsuchida K, et al. Early and late coronary stent thrombosis of sirolimus-eluting and paclitaxel-eluting stents in routine clinical practice: data from a large two-institutional cohort study. Lancet. 2007;369:667-78.

39. Hong SJ, Kim MH, Cha KS, et al. Comparison of three-year clinical outcomes between sirolimus-versus paclitaxel-eluting stents in diabetic patients: prospective randomized multicenter trial. Catheter Cardiovasc Interv. 2010;76:924-33.

40. Kimura T, Morimoto T, Nakagawa Y, et al. Very late stent thrombosis and late target lesion revascularization after sirolimus-eluting stent implantation: five-year outcome of the j-Cypher Registry. Circulation. 2012;125:584-91.

41. Li Y, Li CX, Wang HC, et al. Efficacy and safety of Firebird sirolimus-eluting stent in treatment of complex coronary lesions in Chinese patients: one-year clinical and eight-month angiographic outcomes from the FIREMAN registry. Chin Med J (Engl). 2011;124:817-24.

42. Machecourt J, Danchin N, Lablanche JM, et al. Risk factors for stent thrombosis after implantation of sirolimus-eluting stents in diabetic and nondiabetic patients: the EVASTENT Matched-Cohort Registry. J Am Coll Cardiol. 2007;50:501-8.

43. Palmerini T, Dangas G, Mehran R, et al. Predictors and implications of stent thrombosis in non-ST-segment elevation acute coronary syndromes: the ACUITY Trial. Circ Cardiovasc Interv. 2011;4:577-84.

44. Park DW, Flaherty JD, Davidson CJ, et al. Prognostic influence of diabetes mellitus on long-term clinical outcomes and stent thrombosis after drug-eluting stent implantation in asian patients. Am J Cardiol. 2009;103:646-52.

45. Pinto Slottow TL, Steinberg DH, Roy PK, et al. Observations and outcomes of definite and probable drug-eluting stent thrombosis seen at a single hospital in a four-year period. Am J Cardiol. 2008;102:298-303.

46. Cosgrave J, Melzi G, Corbett S, et al. Comparable clinical outcomes with paclitaxel- and sirolimus-eluting stents in unrestricted contemporary practice. J Am Coll Cardiol. 2007;49:2320-8.

47. Freixa X, Almasood AS, Khan SQ, et al. Choice of stent and outcomes after treatment of drug-eluting stent restenosis in highly complex lesions. Catheter Cardiovasc Interv. 2012.

48. Naidu SS, Krucoff MW, Rutledge DR, et al. Contemporary Incidence and Predictors of Stent Thrombosis and Other Major Adverse Cardiac Events in the Year After XIENCE V Implantation: Results From the 8,061-Patient XIENCE V United States Study. JACC Cardiovasc Interv. 2012;5:626-35.

49. Sardi GL, Maluenda G, Torguson R, et al. Impact of diabetes mellitus on long-term clinical outcomes of patients on chronic hemodialysis after percutaneous coronary intervention. J Interv Cardiol. 2012;25:147-55.

50. Tahara S, Bezerra HG, Kyono H, et al. Impact of acute gain on clinical outcomes of patients treated with sirolimus-eluting stent. - A sub-analysis study from the STLLR trial. Circ J. 2011;75:2113-9.

51. Agema WR, Monraats PS, Zwinderman AH, et al. Current PTCA practice and clinical outcomes in The Netherlands: the real world in the pre-drug-eluting stent era. Eur Heart J. 2004;25:1163-70.

52. Akin I, Bufe A, Schneider S, et al. Clinical outcomes in diabetic and non-diabetic patients with drug-eluting stents: results from the first phase of the prospective multicenter German DES.DE registry. Clin Res Cardiol. 2010;99:393-400.

53. Lemos PA, Serruys PW, van Domburg RT, et al. Unrestricted utilization of sirolimus-eluting stents compared with conventional bare stent implantation in the "real world": the Rapamycin-Eluting Stent Evaluated At Rotterdam Cardiology Hospital (RESEARCH) registry. Circulation. 2004;109:190-5.

54. Marzocchi A, Saia F, Piovaccari G, et al. Long-term safety and efficacy of drug-eluting stents: two-year results of the REAL (REgistro AngiopLastiche dell'Emilia Romagna) multicenter registry. Circulation. 2007;115:3181-8.

55. Singh M, Gersh BJ, McClelland RL, et al. Predictive factors for ischemic target vessel revascularization in the Prevention of Restenosis with Tranilast and its Outcomes (PRESTO) trial. J Am Coll Cardiol. 2005;45:198-203.
